# Caregiving fathers and the negotiation of crossroads: Journeys of continuity and change

**DOI:** 10.1111/1468-4446.12980

**Published:** 2022-10-24

**Authors:** Paul Hodkinson, Rachel Brooks

**Affiliations:** ^1^ University of Surrey Guildford UK

**Keywords:** caregiving, family, fathers, gender, lifecourse, parenting

## Abstract

This article addresses the sustainability of extensive paternal involvement in caregiving by applying a temporal approach to understandings of the journeys of primary‐ and equal‐carer fathers and their families. Drawing on a second phase of interviews with UK fathers first spoken to 5 years earlier, we explore continuities and changes in their roles, identities and outlooks as their caregiving journeys progressed and their children became older. Specifically, we explore how fathers and their families negotiated significant moments of change that had the potential to prompt reflection or reorientation, moments that we term *caregiving crossroads*. Through doing so, we highlight how, in spite of shifts in their arrangements and understandings, the embeddedness of fathers' caregiving routines, bonds and identities had apparently enabled their commitment to counter‐normative roles to endure through such crossroads. We also outline, however, how gendered limitations to some aspects of the scope of their caregiving seemed also to persist as their journeys as fathers progressed.

## 1 INTRODUCTION

The caregiving involvement of fathers has been subject to much recent attention. As part of this, a body of research on the minority of fathers who take on primary care roles or share care equally with partners has begun to emerge (see below), enhancing understanding of their experiences and challenges. Much of this work, though, focuses on caregiving fathers' practices and identities at a fixed moment in time, typically in the first few years of children's lives. Policy conversations tend also to focus on facilitating fatherly involvement soon after their children are born (through encouraging paternity leave, for example), with rather less attention to whether and how extensive fatherly care roles may develop, or recede, over time.

In this paper we begin to address this gap by examining the ongoing development of care roles by UK primary and equal carer fathers, reporting on a recent second phase of interviews with men first spoken to in 2016 (see Brooks & Hodkinson, 2020). We focus here on how the fathers' unusually extensive care roles developed as their children became older, and as changing circumstances were negotiated. We center our investigation on what we term *caregiving crossroads*: significant events, developments or changes that occur within the journeys of caregivers which may prompt arrangements to be reassessed or changed. We focus on three crossroads moments that arose repeatedly in the fathers' accounts—the arrival of another baby, children starting school, and COVID19 lockdown. We explore how the sharing of care was maintained or challenged as these potential turning points were negotiated, assessing the durability of fathers' extensive care roles, the ways they developed and ongoing challenges that were faced.

Our discussion highlights how the men's extensive caring practices, responsibilities and identities seemed to have endured through moments of change, indicating that, once firmly established in their identities and practices, primary or equal care roles among fathers have significant potential to endure as their children become older. We also demonstrate, however, that some of the gendered challenges that can be faced by caregiving fathers in the early years seem also to persist as their journeys progress, sometimes limiting the scope of their roles. Through highlighting these continuities, alongside the negotiations, developments and adaptions in how caregiving fatherhood is lived out over time, we demonstrate the value of a longitudinal approach in understanding men's experiences of extensive caregiving.

## 2 FATHERS AND CARE SHARING—BACKGROUND

Amidst ongoing shifts in family diversity and aspirations for more egalitarian and democratic relationships (Chambers, 2012), studies indicate that fathers increasingly aspire to have greater involvement in their children's upbringing and more intimate connections with them. Yet in heterosexual couples, mothers tend to remain primarily responsible for caregiving in practice (Christopher, 2012; Dermott, 2008; Miller, 2017; Norman et al., 2014). Progress with respect to paternal care has been greatest in some Scandinavian countries where parental leave policies that include fatherly quotas have encouraged greater equality (e.g., Kaufman & Grönlund, 2021), but even here many fathers continue to be secondary carers (Kitterod, 2016). In the UK, only a tiny proportion of men take extended periods of parental leave through the country's “Shared Parental Leave” scheme (Twamley & Schober, 2018), while women with dependent children remain far more likely to work part‐time than men (ONS, 2018). In spite of shifting attitudes, myriad barriers to greater paternal caregiving persist within and outside families, with ongoing practical and normative pressures on women to prioritize caregiving and on men, to fit care involvement around breadwinning (Burnett et al., 2012; Miller, 2011).

Recent studies on the impact of COVID19 lockdown on family responsibilities suggest a further concentration in caregiving discrepancies during this period. Many fathers took on more hours of childcare than ever before during lockdown (Burgess & Goldman, 2021), but still the additional care burden taken on by mothers seems often to have been greater. Research by Calarco et al. (2021) indicates that unequal pre‐COVID arrangements, and an embedded understanding of mothers as default caregivers, normally resulted in these women taking on the bulk of lockdown childcare responsibilities, often without negotiation (also see Zamberlan et al., 2021).

Against this context, a growing body of research on primary carer fathers and, to a lesser extent, those who share care equally, has sought to uncover how such counter‐normative roles are played out, focusing on the extent of responsibilities taken on, shifts in parental and gender identities and challenges that can be faced (e.g., Brandth & Kvande, 2018; Doucet, 2017; Kelland, 2022; Ranson, 2010; Soloman, 2017; Twamley, 2021). Such work often has highlighted the range of care roles and depth of intimacy exhibited by fathers in such families, showing, for example, how skills and bonds developed through everyday caring have prompted the embrace of nurturing paternal identities centered on affective closeness (Ranson, 2015). The embrace of more nurturing forms of masculinity by such fathers has been highlighted here, dovetailing with Elliot's (2016) formulation of “caring masculinities”, for example, while others have understood the significance of the practices of caregiving fathers through Deutsch's notion of “undoing gender” (2007). Studies have also identified limitations to such fathers' embrace of caring roles and identities, however, including difficulties networking with other caregiving parents (mostly mothers) (Doucet, 2017; Soloman, 2017), limits to the transformation of masculinities (Doucet, 2017), continuing beliefs in “natural” bond children have with mothers (Doucet, 2009), or barriers caused by paternal caregiving roles not being appreciated or accommodated at work (Kelland, 2022).

Emphasis on the transformative potential of fatherly caregiving also emerged from the first phase of our own research on primary and equal carer fathers (Brooks & Hodkinson, 2020). Having often been prompted to take on their roles by unusual circumstances, participants had typically come to embrace wide‐ranging nurturing roles and self‐understandings as *interchangeable* with their partners with respect to their care roles (c.f. Ranson, 2010) and had, in our understanding, become comfortable with caring forms of masculine identity in doing so. Their *fatherly care horizons*—visions of what might be feasible or suited to them going forward—had shifted to encompass an ongoing embrace of caregiving responsibilities and a deepening of the intimate bonds they had developed. Yet we also noted a tendency for fathers to cede responsibility to their partners with respect to some elements of decision‐making—so‐called executive responsibility (Ranson, 2012)—and identified struggles when caring outside the home amidst spaces and networks dominated by mothers. Moreover, there sometimes remained a tendency to regard their children's mother as the “default” caregiver (ibid.). For all the progress made, it sometimes seemed that, were circumstances to change, the families might revert to more traditional roles.

## 3 CARE SHARING JOURNEYS, CROSSROADS AND HORIZONS

It is this question about the resilience of care sharing practices and identities amidst changing circumstances that we address in the current paper, through exploring how the journeys of caregiving fathers develop over time. We are cognizant, here, of a small body of existing work examining changes in paternal care over time, most of which centered on broader samples of fathers. This includes quantitative cohort research looking at mixed cohorts of fathers, such as Hwang and Lamb's analysis of the consistency in divisions of care responsibilities in Swedish households (Hwang & Lamb, 1997) and Norman and colleagues' analysis of shifts in fatherly care during children's first 3 years in the UK (2014). Qualitative longitudinal research of equal and primary carer fathers is scarce, though Andrea Doucet (2017) highlights the potential value of such work in revisiting a handful of the families who took part in her seminal “stay‐at‐home fathers” research 9 years later, highlighting, for example, the cementing of reversed care and breadwinner responsibilities, and men's engagement with different forms of paid labor as children became more independent.

Amidst limited qualitative longitudinal work on primary and equal carer fathers, our discussion is, however, particularly indebted to broader studies of (mostly secondary carer) fathers' journeys (Faircloth, 2021; Miller, 2017; Neal & Davies, 2016; Shirani & Henwood, 2011a, 2011b; Shirani et al., 2012; Tarrant, 2021). Shirani and Henwood interviewed such fathers at three different points, using a biographical approach to highlight shifts in hopes, understandings and orientations (2011a). They identify a contrast between early paternal hopes for increasing care involvement and, later, mixed experiences of greater (though still secondary) involvement and anticipation of the receding of responsibilities as children got older. Work by Anna Tarrant also takes a life course perspective, using biographical interviews with low‐income fathers to explore the origins and development of their approaches to caregiving involvement over time (Tarrant, 2021). Meanwhile, having interviewed secondary carer fathers before and after birth, Miller (2017) spoke to a smaller group again a few years later, outlining developments in care practices and understandings, with particular attention to key events such as starting school, the birth of siblings, changes to paid work and relationship break‐ups.

Such key events are of particular importance to our own discussions, which focus on primary and equal carer fathers specifically. In exploring their trajectories, we examine what we call *caregiving crossroads*: expected or unexpected sets of emerging circumstances that may create the conditions for the revisiting of arrangements, roles and identities (Brooks & Hodkinson, 2020). The reaching of such a point may reflect shifts in individual or family circumstances (changing job, moving house), the arrival of institutional milestones (starting school), or the onset of structural change or other external events (recession, pandemic)—or the intersection of multiple factors. Here we draw on Giddens' (1991) notion of “fateful moments” that may prompt reflection and a change of direction, and also understandings of the contrast between periods of routine and “turning points” where different life pathways may be opened up or closed off (Hodkinson & Sparkes, 1997). The notion of caregiving crossroads also connects with Shirani and Henwood's (2011b) emphasis on the role of secondary carer fathers' life course “disruptions”, whereby present arrangements and “imagined futures” become challenged—and Jorge et al. (2022) highlighting of “crucible moments” in the shifting of parents' networks with one another. The metaphor of the crossroads is, we suggest, of particular value in highlighting the reaching of a point in which different possible directions forward may arise—a decision point liable to prompt reflection, negotiation and, ultimately, either continuation or a change in direction.

Shirani and colleagues' aforementioned notion of imagined futures ties in with a further conceptual theme in this paper—on primary and equal carer fathers' visions of who they are and where they might be heading. Here we use our concept *fatherly care horizons* (Brooks & Hodkinson, 2020) to highlight fathers' visions of what is feasible, appropriate or suited for them, and the role such visions can play in enabling or constraining their caregiving practice. Indebted to Phil Hodkinson and Sparkes' (1997) notion of horizons for action*fatherly care horizons* as reflecting the range of structural, institutional and cultural factors that make up fathers' identities and understandings, not least prevailing norms relating to gender and parenthood and the myriad ways these are reinforced. Yet, crucially, horizons also are affected by the idiosyncrasies of particular lives, and by the shifting circumstances fathers and their families experience over time. In the first phase of our research, many fathers pointed to the importance of unusual circumstances in prompting their horizons first to encompass the possibility of taking on an extensive share of caring. What we examine here is how permanent and steadfast this shift was, and how their horizons developed as their journeys progressed.

## 4 METHODOLOGY

In the project's first phase, 24 interviews were carried out with fathers who regarded themselves as equal or primary carers for children aged three or under, and had made adjustments to work to accommodate their caring (Brooks & Hodkinson, 2020). Our interest was in how shared care arrangements play themselves out in heterosexual relationships where both parents are present, so this was a criterion for the sample, recruited through advertisements placed in off‐ and on‐line locations, including children's centers, nurseries, online parenting communities and social media. We explicitly sought fathers who were spending at least as much time as their partners on caregiving and who had adjusted work to accommodate this. A variety of approaches to care sharing were included, with most working flexibly to facilitate being the main carer or sharing care equally. While studies suggest fathers and mothers can differ in their assessments of domestic labor (e.g., Pew Research Centre, 2015) fathers' answers to extensive questions on the division of responsibilities and their apparent openness to acknowledging limitations reassured us that all were fulfilling a wide range of caregiving activities, and, with one or two exceptions (whose care activities were unusually extensive but not quite equal), doing a similar or greater overall share compared to their partners. The sample was demographically homogenous, dominated by white, middle‐class men, something that limits the scope of our findings. Interview questions centered on how the men had come to take on their care roles; the way everyday tasks were distributed; how different aspects of caring had played themselves out in practice; and the men's developing understandings and identities as caregiving fathers and men.

The second phase of interviews took place 5 years later in 2021 and involved 15 men from the initial study who responded positively to an email invitation. Interviews were carried out during and just after one the UK's periods of COVID lockdown, in winter/spring 2021, via Microsoft Teams. They typically lasted for up to 90 min and centered on participants' reflections on how their care roles had developed as their children had become older and through the various events, circumstances and changes that had occurred since we last spoke to them. The latter included their experience of the COVID‐19 pandemic. All interviews were audio‐recorded and fully transcribed.

Our analytical approach involved both deductive and inductive approaches. We coded the transcripts thematically, drawing on themes and questions relating to gender and caregiving derived from literature (and, for the second wave, key themes developed from the previous wave of interviews), while also developing a range of additional codes to record emerging understandings. In reporting the data below, we refer to participants via pseudonyms (used across both phases of research) to preserve their anonymity. While the discussion incorporates aspects of the findings from both phases of the research, the predominant focus is the second set of interviews and all quotations presented are from this phase.

## 5 FINDINGS: CONTINUITY AND CHANGE THROUGH CROSSROADS

It was clear from the second interviews that fathers had sustained substantial caring responsibilities and a strong commitment to maintaining these. Reflecting on how their lives and roles had developed, though, they spoke of a variety of shifts, changes and challenges, some of which had led to adjustments in how care was organized, or developments in fathers' understandings and care horizons. Some such developments were gradual, such as incremental shifts in approaches to care as children became more independent, an ongoing deepening of bonds as fathers become more experienced and the cementing of an understanding of themselves as caregiving fathers. Conversely, while many had retained a relatively consistent split between paid‐work and care over time, we did identify a gradually expanding awareness about the impact their ongoing commitments to care were having on their careers and, sometimes, anticipation in their horizons of a moment in the future where caregiving responsibilities might ease.

Alongside such gradual developments, a variety of specific events were identified that had brought fathers and their families to crossroads, leading to reflection and, sometimes, the possibility of changes in direction. Including a mixture of individual or family circumstances, institutional milestones and external developments, examples of such events included moving house, changing employment situations and shifts in the availability of day care. The three events that had most significance across interviews, though, were the birth of a new baby, children starting school, and the COVID‐19 pandemic. In the discussion that follows, we explore the significance of these crossroads moments in fathers' journeys, the ways they were negotiated and the changes and continuities they highlighted. Overall, in spite of changes to the detail of caregiving and fathers' understandings, we were struck by the extent to which their embrace of primary or equal care roles and identities had endured. Also enduring, though, had been some of the limitations and challenges to caregiving identified in the first phase of our research.

### 5.1 Crossroads 1: A new baby

For several fathers, the arrival of a new baby had created a substantial crossroads moment. In their first interviews, some had already indicated the birth of a new baby might bring uncertainty over the continuation of their care roles because of the expectation their partner would take exclusive maternity leave. In the second interviews, it was clear that, in every case where a new baby had been born, there had indeed been a substantial transfer of care responsibility back toward the mother during the postnatal period. Ryan, who was sharing care equally with his partner, had had two additional children since his first interview and on each occasion his partner had taken significant periods of maternity leave, with little discussion of the possibility of sharing the leave equitably.

You know, with two children being born since we last spoke, then [partner's name] was off for maternity leave for relatively large chunks of the last four years when I wasn't. So during those times then, yeah, [partner] has been doing nothing but childcare.

(Ryan)

A discussion about paternity leave *had* taken place between Kevin and his partner, who normally shared care equally, but his partner actively wanted to take the full period (12 months) and the baby coincided with him being promoted at work, prompting a traditional post‐natal arrangement:

[Partner] wanted to do the 12 months again, because she really enjoyed it the first time around. At that stage, I was just about to be promoted. So, we had the discussion about it and we, we sort of said, well maybe the final three months… then we could swap… decided it probably didn't fit with where we wanted to be. So [partner] did all the maternity leave.

(Kevin)

Although David was “stay‐at‐home‐dad” in his family, when his second child was born, his wife took 6 months of maternity leave, prompting a shift to a more equal split during this time. He explained that the maternity leave was regarded as a chance for his partner to make up for regrets she sometimes experienced about him spending more time with the children:

I think because [partner] wanted to spend a bit more time… because she, I don't know if she was a bit jealous, I don't know, if that's the right word. Envious maybe? Just of the time that I was spending. But what she was able to do was actually take the full maternity leave.

(David)

While the switch toward maternal care in these and other cases partly reflect practicalities such as post‐birth recovery and breast‐feeding, it also highlights the resilience of post‐partum gender norms. That men who had become comfortable with extensive care roles had ceded such a significant portion of responsibility, and that women used to sharing or breadwinning had switched back to far greater care responsibility for up to a year, underlines the rootedness of gendered expectations on care in the first year after having a baby (see Doucet, 2009). The sudden shift toward traditional gendered roles that entering parenthood for the first time can prompt in couples that aspire to equality has been well established (e.g., see Faircloth, 2021; Miller, 2011). Yet the switch back toward maternal care for fathers in this study is particularly striking because it accompanied the birth of a second or third child, *after* an unusually progressive split had been established in the care of their older siblings.

The data also highlight how the men often felt their partners had experienced guilt at doing a lower proportion of hands‐on caregiving for existing children than is often expected of women, and were keen to use the crossroads of a new baby to redress this. Here, our research indicates that the feelings of maternal guilt women can experience when they return to work after having a baby (Berger et al., 2022; Christopher, 2012) may manifest themselves—and endure—particularly strongly in families where caregiving is shared in counter‐normative ways. Moreover, the emphasis fathers placed on the primacy of their partners' wishes in determining allocation of parental leave illuminates a tendency even for experienced care sharing fathers to continue to regard their children's mother as the person with “first preference” on caregiving responsibility. This ceding of maternal first preference, we argue, forms part of a broader discourse of *default maternal responsibility* that seemed to continue to operate both within and outside the men's families (Brooks & Hodkinson, 2020).

Crucially, however, post‐partum shifts toward maternal responsibility did *not* seem to lead to any noticeable reversal in the fathers' long‐term care responsibilities or their horizons. Fathers appeared to have continued with their own established split between work and home while their partners were off work, and then returned to an approximation of previous distributions of care immediately afterward. Although David's partner took 6 months of maternity leave, he had remained at home and contributed extensively to the care of both new baby and older sibling during this time—making a significantly greater contribution than he had for their first baby. After the leave was over, his partner returned to full‐time work and he to his role as primary carer. James had continued to take Wednesdays off work during his partner's 12 months of maternity leave, offering support with the new baby but focusing particularly on their first child. When his partner returned to work from maternity leave, he cared for both children on Wednesdays and routines reverted largely to the sharing arrangement they had had before.

My wife had that whole‐ she took a whole 12 months off for maternity leave … other than that, so all our arrangements are largely the same as they were, just with two children rather than one… I still stayed on the same not working Wednesdays [during maternity leave]… And then after my wife went back to work… I would just be doing things with both children.

(James)

Overall, then, the birth of a new baby acted as a caregiving crossroads in the sense that it prompted reflection and re‐assessment of care arrangements. It created a potential turning point where pressures to revert to doing gender in more traditional ways came to the fore and highlighted how notions of default maternal responsibility had continued to linger in the background for both fathers and their partners. It also indicates, however, that the fathers' care routines and horizons had become sufficiently entrenched that a reversion back to counter‐normative care sharing once their partners returned to work seemed to take place with minimal doubt or negotiation. The switch back to maternal care in the post‐natal period, then, seemed to have been temporary.

### 5.2 Crossroads 2: Adjusting to school

The moment that children started school also had acted as a significant crossroads moment, where routines were subject to reflection and responsibilities had the potential to shift. Although some of the families had used day‐care on certain days, full‐time school represented an end to the need for all‐day parental care during the working week, while symbolically closing the intensive period of early‐years care. At the same time, school created new drop‐off and pick‐up arrangements and the need to arrange or administer care from mid‐afternoon. As well as its significance with respect to care routines and horizons, we explore here the importance of the transition to school‐centered life in development of some of the barriers caregiving fathers had experienced in the early years.

Not surprisingly, most of the fathers described changes to the detail of their everyday care routines, which became focused on preparing and taking children to school and looking after them from mid‐afternoon (normally), while adjusting to child‐free time during school hours. In most cases, though, fundamental shifts in the distribution of care responsibility were hard to identify. Fathers who worked flexibly to have days off during the week seemed either to have retained these days‐off or converted them into after‐school hours across more days. John, for example, had converted his 4 days of work into 5 shorter days. If anything, there were one or two signs that early‐years gendered pressures to care experienced by mothers may sometimes have receded a little with the onset of school. For Brian, the distribution of care between himself, his partner and their children's grandparents had been largely replicated with respect to responsibility for after school care—but he also felt that the transition to school seemed to have helped assuage some of the anxieties his partner had experienced during the early years and ended a previously‐mooted possibility of her going part‐time to become primary carer:

It's just, I guess in those early stages as a parent, particularly I think as a mother, you have all these sorts of conflicting guilts coming at you and, yes, I think there was part of her that thought that she should keep her mind open to it [going part‐time]. But I think because we've got a situation where… she, [child's name]'s at the local school. It's literally down the road from us. It's not as though we are ever in a position where we feel we're missing out on time with [child's name]. So I think that's kind of, that's probably what side‐lined that issue.

(Brian)

While the balance between caregiving and work seemed not to have been substantively affected by the shift to school life for working fathers in the sample, the crossroads seemed more significant for those who had given up paid‐work during the pre‐school years. As time had progressed, David's passion for his role as stay‐at‐home‐father had started to become offset by worries about his career being on hold. A gradual shift in his horizons toward a desire for balance between caregiving and career transformed into an active quest for work in the run‐up to his children beginning school and day‐care respectively. Institutionalization of day‐time care, then, had become a significant crossroads, opening up the possibility find a part‐time job in the mornings, with his partner rearranging her own hours to cover care and drop‐off in the mornings. While this had resulted in a shift toward a more shared care arrangement, David's overall commitment to caregiving remained extensive: he turned down the chance to work full‐time to ensure his afternoons were free to care for the children, and his relief at having found employment that enabled such balance was clear:

… they were like, well, we can get you up to full time by offering you this, this and this. And I was like, actually, it works really really nicely for me the way it is… I was happy being back in… getting back to something that I enjoyed, and I knew I was quite good at… And also, there was a, I think there was an element of relief there. That I've been able to find something and the hours worked.

(David)

The onset of school, then, seemed sometimes to have allowed for a shift in the direction of work in the horizons of stay‐at‐home fathers. This may partially reflect the particular pressures men can face with respect to career and breadwinning (Burnett et al., 2012), but also bears at least some comparison with the pathways and dilemmas of mothers who relinquish paid work through their children's early years and feel pulled back toward the labor market with the onset of school (Yahraes, 2017). Crucially, however, the crossroads created by children starting school had not led to significant changes to the distribution of care in most of the families, or to shifts away from fathers' embrace of unusually extensive care roles.

The transition to school also marks a potential shift in the broader parenting environment in which caregiving fathers operate. Studies have identified early years networks of institutions and parents as highly mother‐centric, creating environments that fathers can find it challenging to engage with (Doucet, 2017; Soloman, 2017). In their first interviews, many of the fathers highlighted gender‐related difficulties with such pre‐school environments, from professionals preferring to communicate with their children's mothers, to broad feelings of social discomfort in daytime parenting spaces (Brooks & Hodkinson, 2020). Unfortunately, while some indicated that they had become a little more comfortable interacting with professionals and other parents, for most it seemed that their pre‐school sense of marginalization had reproduced itself—at least to some degree—in school‐centered routines and environments. Several fathers observed that schools themselves had a tendency to seek communication with their partners rather than themselves, even in those cases where they themselves were listed as first contact, as in Anthony's account below:

…they have a preference, I think, to deal with the mother… we've noticed it at both the school and the nursery. It was a bit weird with the school… I went and filled out all the paperwork… so I put my details first… but they've always only ever contacted [partner name], you know, she still has to forward all the emails on to me… It's less apparent than it was say with health visitors and midwives and all that. But… that's an external thing that's placed upon us.

(Anthony)

As well as having less direct communication with schools themselves, fathers tended to be relatively disconnected from networks of other parents. Though some felt the difficulties were not as stark as they had been at playgroups or “mother and baby” events, most spoke about how they had found it difficult to engage with school‐centered parent networks, either at school gates, or via parents' WhatsApp groups, which had become key sources of information and typically involved few if any fathers. Like some others, Brian saw his own “introversion” as a factor in his partner doing most of the communication with other parents, but also felt that gender barriers were continuing to play a role, contrasting what he saw as mothers' tendency to converse in groups with the isolation of fathers at the school gates:

I don't know what it is, but there seems to be this kind of invisible barrier between mums and dads, and you'll see mums instinctively go out of their way to talk to other mums, but they don't tend to do it with dads… I often find it's a situation where there's, there's lots of mums, and they're all chatting to each other… and then there'll be two or three dads all just sort of stood in silence sort of on their own.

(Brian)

The precise workings of such gender dynamics in communities of parents warrant more detailed attention, not least with respect to the extent of the role of mothers and/or fathers, as well as institutions such as playgroups, nurseries and schools in their reproduction (see Brooks & Hodkinson, 2020). Suffice to say that, though some of the fathers felt the situation had improved a little as their children had become older, the gendered social barriers they experienced seemed still to be preventing some from participating fully in what Doucet (2017) calls “community responsibility” aspects of parenting, including the organization of playdates and parties, as Robert shows:

She typically has a closer relationship with those women than I do. Which I guess is just because, I don't know, friendships tend to be quite gender‐linked… So the net result is it's always a bit easier for her to be doing the contacting… And I think I just I'm a bit more reluctant to contact people and say, “would you like to go on a play date” even if it's like my day—I'm more inclined to say, I'll just look after the kids by myself.

(Robert)

As a result, communications with other parents—and sometimes with school—had a tendency to be left to the men's partners and there were few signs the early years challenges fathers faced with respect to these had been relieved. For all the responsibilities both taken on and sustained elsewhere, this meant there remained gaps in the sharing of care when it came to these key organizational matters, something that can be connected to broader discourses of default maternal responsibility that were reinforced by the institutions and parenting environments in which fathers operated and, sometimes, by their own understandings and actions. Elsewhere, we explore how this enduring challenge in caregiving fathers' journeys connects to a broader tendency for educational organizational and decision‐making responsibilities to fall more heavily on the men's partners than themselves (Brooks & Hodkinson, 2022).

In summary, then, while it created a clear crossroads moment for fathers and their families that was typically negotiated through changes to routines and—sometimes—a greater emphasis on paid work, children starting school did not seem to make a substantive difference to fathers' commitment to caregiving or the broader sharing of care in most of the families. But neither did it seem to alleviate previous struggles interacting with institutions and other parents.

### 5.3 Crossroads 3: Care sharing in a pandemic

While the first two case studies focus on key events that are a usual part of parenting journeys, the COVID19 pandemic created a once‐in‐a‐generation set of externally‐driven changes and challenges for the families. Given the extensive evidence emerging about the disproportionate additional caring burden taken on by mothers during the pandemic (Calarco et al., 2021), we were interested to see, for the families in our sample, whether the demands of lockdown^1^ and the situation of both partners and children potentially being ever‐present in the home, might prompt a turn back toward more traditional roles.

Contrary to some of our conjecture, however, there was little evidence of any shift back toward maternal primary responsibility and, in most cases, fathers had taken on as much or more care responsibility than beforehand. Practical expediency and interchangeability had formed an important driver of many of the families' shared care arrangements in the past, and a similar approach had often been taken in lockdown, especially with respect to work requirements. Where both partners had an equal load with respect to their employment, the extra caregiving that lockdown required seemed to have been split fairly evenly, reinforcing existing equal sharing arrangements. In several cases, though, differences in work arrangements had resulted in fathers taking on a greater proportion of the family's care burden than previously. This sometimes reflected specific lockdown arrangements, such as the mother having to continue to go to work while the father remained at home, but also related to a broader tendency for fathers to regard their partners' careers as more important than their own—something identified consistently in the first wave of interviews. Moreover, the embedding of paternal caregiving practices, relationships and horizons over time meant that these fathers found themselves particularly well‐positioned to take on greater responsibility when faced with such unusual circumstances.

Kevin and his partner had established a long‐term shared caring arrangement where each had prioritized career or caregiving at different points, and the onset of COVID19 had precipitated one among many shifts in their arrangements. They had moved to another country for Kevin's partner's job just before the pandemic and, because of the importance of this for her career, he had drastically reduced his own working hours during lockdown, with the majority of his days spent caring and home‐schooling:

it was really important to us that she [partner] could maintain the integrity of the work she was doing… so she was going into work most days… But it meant that, my work stopped… And so our pattern shifted, [partner] still did nine 'til five… And then throughout the day, I did nothing because I had the children. And even when Zoom schooling came in for our older one, I still had our younger daughter who still wants a lot of attention. So the balance shifted a lot during COVID.

(Kevin)

John had also switched to taking on most of the home‐schooling during the first UK lockdown because his contract at work was coming to an end, giving him greater flexibility at the time than his partner, whose career he felt should be prioritized. However, by the time schools were closed again in January 2021, John was coming to terms with a busy, high pressure new job of his own, while his partner had taken on a strategic role that entailed greater flexibility. As a result, they decided that home schooling would be shared equally this time, with ongoing juggling of responsibilities:

So the first the first time around… once it became obvious the situation we were all in, I was like, right, I'll just do it [home schooling]. And my wife was happy with that knowing my work situation. And yeah, this time round, it has just been a balancing act. It's just been an ad hoc thing… either we will sit down and look at each other's diaries and say, Okay, can I do the morning? On that day? Yeah, that's fine. Or I've got to do the morning on that day. Yeah. Okay… And then then we might have to tweak it… It's just about what we've got on.

(John)

Such examples are striking in their contrast with the disproportionate lockdown burden on mothers in families with more conventional pre‐lockdown care roles (Calarco et al., 2021). They show how routines, skills and horizons centered on care sharing and interchangeability had established themselves in the understandings and horizons of these fathers and their partners, both of whom were negotiating their respective work constraints to take‐up different responsibilities as circumstances developed. Rather than a reversion back to traditional roles, what we saw were arrangements and principles established through years of sharing care becoming further reinforced when the crossroads moment of lockdown was encountered. These fathers found themselves particularly well‐placed and willing to take on an equal or greater share of care during lockdown, then, because such an eventuality already lay within their skillsets and horizons.

As part of their lockdown responsibilities some fathers had also made limited inroads into some of the barriers alluded to in the previous section. Some had taken on significant communication with teachers and school during the period, for example, or engaged in communication with other parents—and most had some degree of involvement with the organization of lockdown care and home schooling as well as its delivery. James, who seemed to have been doing slightly less caregiving than his partner up to that point, had taken on most of the home schooling throughout the week during lockdown because her work had intensified while his allowed greater flexibility. It was clear this included involvement in organizational aspects of care, including developing a first draft of their daily timetable. He emphasized how rewarding he had found the experience of expanding his care responsibility during this time:

So I was probably doing the majority of it. Certainly on a Monday, Wednesday, and Friday because my wife's job requires her to be focusing and paying attention to what she's doing. Whereas I was lucky enough to be able to sort of have one eye on the computer and one eye on the kids… We made it deliberately not too intense… it would be, we'll do an hour, and then we'll do something completely different, and then we'll do another hour of something… I think I put together the first draft of here's an idea for a timetable, and we altered it accordingly.

(James)

Notwithstanding encouraging signs such as these, it remains our broader impression that barriers to fathers' communication and organizational involvement often had persisted. In particular, progress in overcoming a tendency identified in our original study for mothers to take on greater “executive responsibility” for care (Ranson, 2012) seemed partial at best, with mothers still extensively involved in the coordination of children's learning. Even where fathers took on greater everyday organizational tasks, there often seemed little change to mothers' responsibility for medium‐ or longer‐term decisions. Kevin outlined how he felt that his partner had always been more active in researching and planning their children's development and, even with the shift to him doing the majority of childcare during lockdown (including aspects of liaison with schools), she had remained heavily involved in setting the direction of caregiving, researching activities he could do with the children, buying books and contributing to routines:

I think, that kind of goes back to [partner] being that kind of person who's always thinking about what else can we do to develop children, so she'd always implant ideas into my mind. Or she'd start an activity one evening with the girls and say, oh, you can carry on this with daddy tomorrow. So it very much becomes part of us both sharing those kind of responsibilities. [partner] still… like pre COVID, and COVID, [partner]'s still very much involved in what we kind of do and how we do that, even though she's at work most of the time…

(Kevin)

In one sense, then, Kevin had taken on greater levels of everyday organizational responsibility as a result of the crossroads created by lockdown, but on another, the extent of his partner's input into research and planning indicates a continuing sense of executive responsibility on her part, something replicated for several others. As we have argued elsewhere, it seemed that, while fathers had taken on extensive roles as educational laborers during lockdown, their partners often seemed to retain responsibilities as “educational executives” (Brooks & Hodkinson, 2022), reflecting, among other things, a continued compulsory maternal burden of moral, mental responsibility, even where men take an equal or greater share of practical caregiving (Christopher, 2012; Faircloth, 2021; Miller, 2017).

While lockdown had created a significant crossroads, then, it had not noticeably resulted in a return to more traditional care arrangements. Rather, fathers' existing familiarity with the routines and principles of care sharing had enabled either a continuation or concentration of fatherly caregiving. Likewise, though, in spite of some signs of change, the responsibility on the men's partners with respect to organization, decision making and moral responsibility seemed to have persisted.

## 6 DISCUSSION—CARE SHARING CONTINUITIES

This article incorporates a temporal dimension to our understanding of paternal caregiving (Shirani & Henwood, 2011b) in families where fathers discharge extensive and counter‐normative care roles. By understanding the journeys of such fathers and their families over time we can assess the long‐term durability of such arrangements, and the ways different sorts of challenges are negotiated over time, in the context of ongoing debates about increasing family diversity (Chambers, 2012) and the uneven development of more nurturing, caring fatherhoods and masculinities (Elliott, 2016). We have highlighted, in particular, the importance of what we term *caregiving crossroads*, moments at which arrangements, roles and identities that have become routine may be revisited or disrupted, and their potential to become turning points in the journeys of caregiving fathers. Drawing on work on careers and the life course (Hodkinson & Sparkes, 1997) we pointed to the importance of *fatherly care horizons* in understanding how what fathers can see as possible or feasible in their visions of themselves and their futures, can affect the kinds of practices and responsibilities they are able to take on, develop or continue.

Through examining three case studies of caregiving crossroads that repeatedly came up in our second phase of interviews, we have explored how such moments are negotiated. Our analysis shows that, while these crossroads prompted reflection, reassessment and change—and sometimes enabled the resurfacing of normative pressures—it was the continuities in fathers' caregiving roles and identities through such moments that were most striking. Although temporary reversions to maternal post‐natal care highlighted the endurance of deep‐seated mother‐centric understandings of the first year of baby's lives (Doucet, 2009), and fathers' tendency to cede first preference on care responsibility to their partners, this maternal switch had in each case proved temporary. While children beginning school marked a significant moment that affected the detail of caregiving routines and, sometimes, a shift of the orientation or routines of some fathers (and mothers) in the direction of employment, we saw no signs of a reversion back to secondary care roles among the fathers. Most strikingly, the dramatic onset of COVID19 lockdown had typically been responded to with a concentration of counter‐normative caregiving divisions that already had been established rather than their reversal.

The years during which these fathers had carried out and established themselves in primary or equal care roles had, we argue, enabled them to negotiate these and other crossroads, retaining and sometimes extending their caregiving practice, responsibilities and horizons in the face of challenge and change. The skills, relationships and affective ties developed through the ongoing practice of care—and particularly through caring for children alone (Brandth & Kvande, 2018; Ranson, 2015)—had apparently cemented their comfort with and commitment to their roles and the ongoing development of horizons that encompassed the long‐term sharing of responsibilities and a sense of interchangeability with their partners (Brooks & Hodkinson, 2020). Thus, in the same way that, for Calarco et al. (2021), the establishment of mother‐centered care for most families over many years had resulted in a continuation or concentration of this in lockdown, so the counter‐normative roles and horizons long established within the families in our sample seemed to enable these to persist, or even intensify through moments of change or challenge.

This overall continuity in their roles and horizons as carers did not mean, though, that certain gendered ceilings on the fathers' caregiving had disappeared. Rather, practical and discursive limitations that had first emerged when their children were younger seemed to have largely persisted. Fathers continued to face barriers communicating with institutions and networks of other parents, hampering their deployment of key organizational and community aspects of caregiving (Doucet, 2017). And there remained a broader enduring tendency for mothers to take greater responsibility for key aspects of decision making, or executive responsibility even where the father carried out most everyday care. The ongoing significance of such constraints highlights, we argue, the durability of discourses of default maternal responsibility both outside and within the fathers' families. Such gendered understandings, which continue to construct mothers as having an assumed first responsibility for care, informed the approach of institutions and other parents in their communication with the families, and sometimes manifested themselves internally, through fathers' tendency to regard their partners as having “first preference” on parental leave—and an ongoing tendency for their partners to feel “pulled toward care” (Doucet, 2009), especially with respect to coordination—part of what Christopher calls, “extensive mothering” (2012).

Through highlighting these contrasting continuities in fathers' care practices and horizons through moments of turbulence—and through detailing the negotiations, shifts and developments such moments can engender—this paper highlights the importance of attending to the temporal development of the journeys of primary and equal carer fathers. Though based on a relatively small and homogenous sample of UK caregiving fathers, such an approach also offers insights of significance for policy‐makers and practitioners. The resilience of discourses of default maternal responsibility through the fathers' journeys highlights a need to address such understandings and the practical barriers they can create. Notably, schools and other institutions of importance in the journeys of parents and families might prioritize the inclusion of fathers in their communications with families and support their involvement in school‐based parent networks. Principally, however, our evidence on how durable counter‐normative care arrangements proved to be once routines and horizons had been established underlines the value of policies that support and encourage the early adoption of care sharing by fathers, not only in the form of more effective paternity leave policies, but also the development of greater support for flexible working among fathers. For our evidence suggests that, if fathers can be supported and encouraged to become heavily involved in early‐years care, this has the potential to set in place routines, skills, bonds and horizons that can endure.

## Data Availability

Research data are not shared.

## ENDNOTE

^1^
The UK government imposed three periods of lockdown, March 26–July 4, 2020, October 31–December 2, 2020 and January 6–March 8, 2021. Only the first and third involved school closures.
